# Contactin-associated protein-2 and anti-aquaporin-4 antibody positive autoimmune encephalitis secondary to herpes simplex encephalitis: A case report

**DOI:** 10.1097/MD.0000000000033767

**Published:** 2023-05-17

**Authors:** Lin-Ming Zhang, Huan-Bo Zhang, Yong-Fang Zou, Ming-Wei Liu

**Affiliations:** a Department of Neurology, the First Affiliated Hospital of Kunming Medical University, Wuhua District, Kunming, China; b Trauma Center, the First Affiliated Hospital of Kunming Medical University, Wuhua District, Kunming, China; c Department of Emergency Medicine, the First Affiliated Hospital of Kunming Medical University, Wuhua District, Kunming, China.

**Keywords:** anti-aquaporin4 antibody, anti-contactin-associated protein-2 antibody, autoimmune encephalitis, case report, herpes simplex encephalitis, human herpes virus type I

## Abstract

Recurrent herpes simplex encephalitis (HSE) can easily induce autoimmune encephalitis (AE). However, there are few reports of anti-contactin-associated protein-2 (CASPR2)-related encephalitis, especially with positive anti-aquaporin 4 (AQP4) antibodies.

**Patient concerns:**

A 14-year-old boy was admitted to the Department of Neurology of the First Affiliated Hospital of Kunming Medical University for “headache, dizziness, and fever for four days” with positive anti-CASPR2 and anti-AQP4 antibodies in the cerebrospinal fluid.

**Diagnoses:**

Cranial MRI showed lesions in the right hippocampus, amygdala, and insular lobe, with local sulcus enhancement in the right insular, temporal, and frontal lobes. The fluid-attenuated inversion recovery was significantly enhanced. Human herpes virus type I was detected by cerebrospinal fluid metagenomic testing. The patient was diagnosed with AE secondary to HSE, with positive anti-CASPR2 and anti-AQP4 antibodies.

**Interventions:**

After 2 weeks of immunoglobulin and methylprednisolone immunomodulatory therapy, acyclovir antivirus, mannitol dehydration, reducing intracranial pressure, and other symptomatic support therapy.

**Outcomes:**

The patient’s symptoms significantly improved, with no complaints of discomfort, and he was discharged for observation. The patient was followed up a month after discharge and had no complaints of discomfort.

**Lessons:**

CASPR2 and anti-aquaporin-4 antibody-positive AE have not been reported to be positive. This case will raise awareness of CASPR2 and anti-aquaporin-4 antibody-positive AE secondary to HSE, strengthen diagnostic capacities, and provide advice to treat it.

## 1. Introduction

The herpes simplex virus invades the central nervous system (CNS) and causes corresponding inflammatory changes, clinically known as herpes simplex encephalitis (HSE) or acute necrotizing encephalitis. It primarily invades the temporal lobe, frontal lobe, and the limbic system. It is the most prevalent viral infection in the CNS.^[1]^ The disease incubation period is between 2 and 21 days, and fever, general discomfort, diarrhea, and other symptoms may occur in the prodromal period.^[1]^

Autoimmune encephalitis (AE) is caused by an autoimmune process. AE is closely associated with viral infections. HSE recurrence is a crucial factor inducing AE, primarily affecting the CNS.^[2,3]^ Although many autoimmune encephalitides have been identified globally, studies of anti-contactin-associated protein-2 (CASPR2)-related encephalitis are rare. This is the first reported case of AE with positive CASPR2 and AQP4 antibodies on the basis of HSE.

## 2. Case report

### 2.1. Ethics approval

Informed written consent was obtained from the patient and his parents for the publication of this case report and accompanying images. This study was reviewed and approved by the local ethics committee of the First Affiliated Hospital of Kunming Medical University. The procedures were in accordance with the Helsinki Declaration of 1975, as revised in 2000.

A 14-year-old male was admitted to the Emergency Department of the First People’s Hospital of Kunming, considering “upper respiratory tract infection” due to “headache and dizziness after catching a cold and persistent pain in the parietal and occipital, with fever, nausea, and vomiting stomach contents for 4 days. The highest body temperature was 40°C. The symptoms of infusion treatment, such as “ribavirin and Yanhuning,” did not improve, and he visited the Department of Emergency of the First Affiliated Hospital of Kunming Medical University on March 3, 2021. Considering the cause of the fever and central infection, he was admitted to the Department of Neurology of the First Affiliated Hospital of Kunming Medical University on March 3, 2021. The patient was diagnosed with Guillain–Barre syndrome in the Department of Neurology of the First Affiliated Hospital of Kunming Medical University on June 13, 2020, and was treated with gamma globulin 2 g/kg for 5 days, the patient’s condition improved and discharged.

Physical examination included somnolence with a GCS score of 15 (eye-opening: spontaneous, 4; language: normal, 5; exercise: obeying instructions, 6), fluent speech, physical examination cooperation, normal orientation, calculation, memory, neck ankylosis, negative Kernig sign, negative Brudzinski sign, left upper limb muscle strength: 5-grade, and left upper limb tendon reflex: (+++). Other limb muscle strength and muscle tension were normal, and pathological signs were negative. The limb pain, temperature, touch, pressure, position, and vibration were normal.

**Figure 1.**
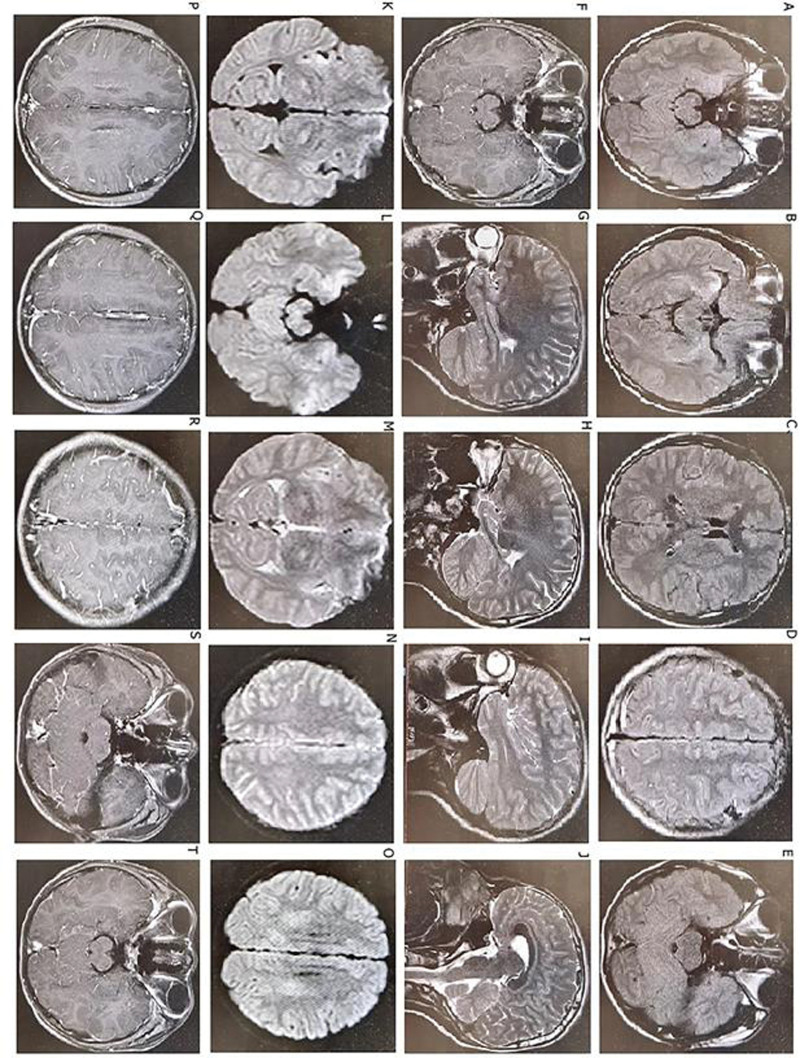
The results of cranial MRI (plain scan + enhanced + MRA + MRV). The findings revealed that the lesions of the right hippocampus, amygdala, and insular lobe and local sulcus enhancement of the right insular, temporal, and frontal lobes were enhanced using FLAIR. (A–E) T2 FLAIR image. (K–O) Diffusion-weighted image. (F–J and P–T) T1-weighted image. FLAIR = fluid-attenuated inversion recovery, MRA = magnetic resonance angiography, MRV = magnetic resonance venography.

**Figure 2.**
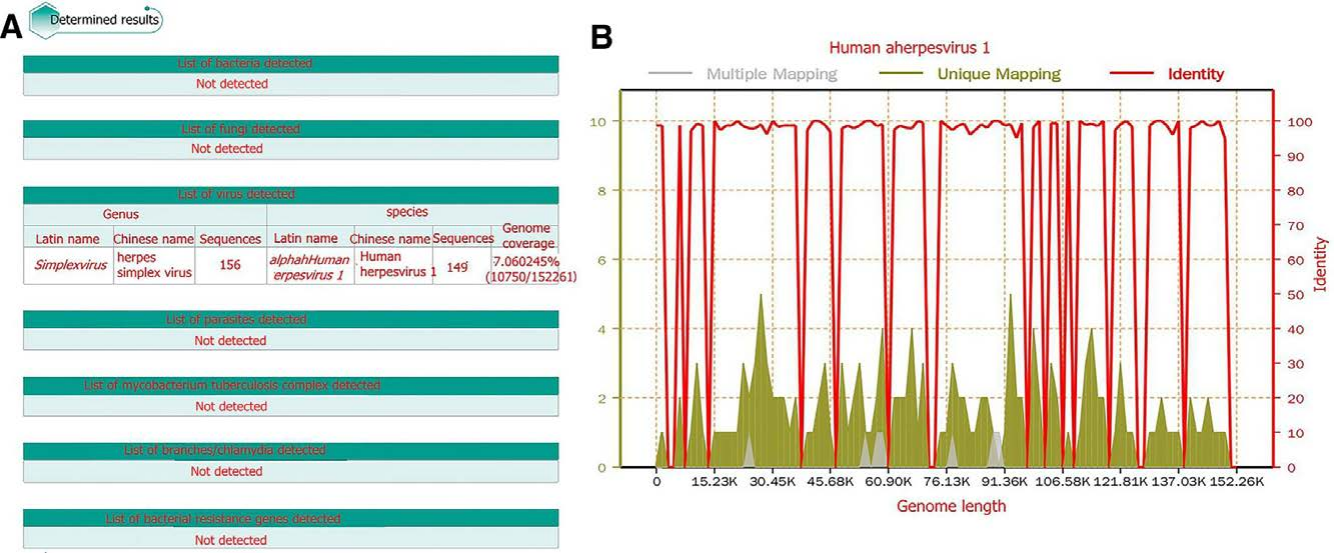
Results of cerebrospinal fluid and metagenomic detection of pathogenic microorganisms. (A) Results of macrogenomic detection of pathogenic microorganisms. (B) Genomic coverage map of human alpha herpes virus 1 (HSV1) in cerebrospinal fluid of the patient. The total base number of the genome of HSV1 is 152,261 (bp). The total length of the sequence covered by the patient is 10,750 (bp), the coverage is 7.060225%, and the average depth is 1.09X.

**Figure 3.**
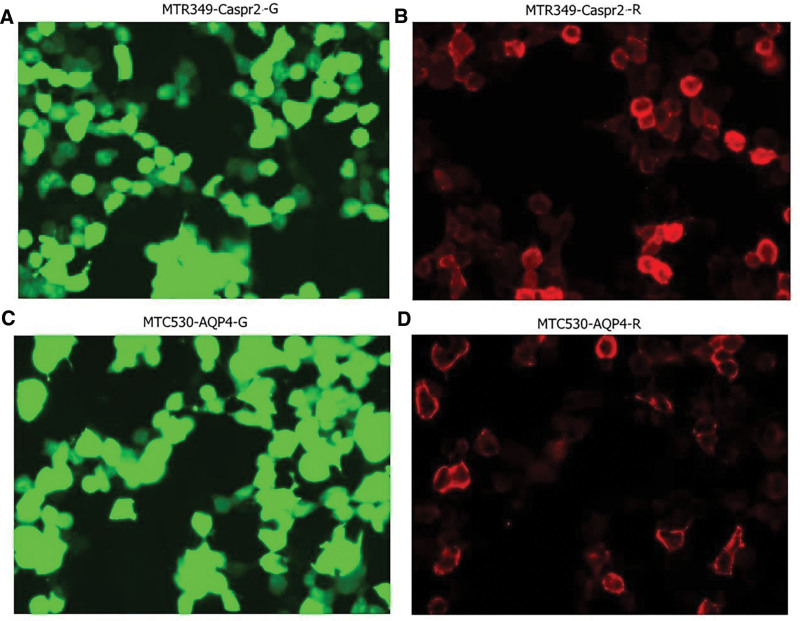
Positive results of antibodies associated with autoimmune encephalitis in cerebrospinal fluid and demyelinating disease of the central nervous system. The positive results of autoimmune encephalitis-related antibodies and central nervous system demyelinating disease-related antibodies in cerebrospinal fluid were detected using immunofluorescence. (A and B) Serum anti-CASPR2 antibody IgG positive (+) 1:10. (C and D) anti-AQP4 antibody (+) 1:10. AQP4 = aquaporin 4, CASPR2 = contactin-associated protein-2.

Laboratory data: As of March 3, 2021 – complete blood count: white blood cell, 4.85 × 10^9^/L; N, 78%; red blood cells, 4.95 × 10^12^/L; hemoglobin, 142 g/L, platelet, 215 × 10^9^/L; procalcitonin < 0.05 mg/L; C-reactive protein, 6.8 pg/L. Chest computed tomography: no obvious abnormalities were found in the lungs. Skull MRI (plain scan + enhanced + magnetic resonance angiography + magnetic resonance venography): lesions in the right hippocampus, amygdala, and insular lobe. Local sulcus enhancement in the right insular, temporal, and frontal lobes was significantly enhanced on fluid-attenuated inversion recovery (Fig. 1), and meningoencephalitis was considered. As of March 4, 2021, the cerebrospinal fluid was transparent, colorless, positive in Pan test (−), white blood cell count 332 × 10^6^/L, neutrophil 5%, red blood cell count 1 × 10^6^/L, no Cryptococcus on cerebrospinal fluid ink staining, and no fungal spores or hyphae on cerebrospinal fluid fungal images (cerebrospinal fluid biochemistry). Glucose: 3.52 mmol/L, chlorine: 125.10 mmol/L, trace total protein: 0.40 g/L, adenosine deaminase: 0.5 u/L, and lactate dehydrogenase: 30 u/L. A set of cerebrospinal fluid macrogene tests of pathogenic microorganisms showed herpes simplex virus (identified sequence number 156) and human herpes virus type 1 [identified sequence number 149, genome coverage 7.060245% (10750/152261)] (Fig. 2), suggesting human herpes virus type 1. On March 7, 2021, AE-related antibodies were detected by immunofluorescence. The findings revealed that the serum anti-CASPR2 antibody IgG was positive (Fig. 3A and B). The serum anti-AQP4 antibody was positive using the CNS demyelinating disease-related antibody test (Fig. 3C and D), while AE and CNS demyelinating disease-related antibodies were negative. Tuberculosis, systemic lupus erythematosus, and rheumatoid-related antibodies were tested and the results were negative.

Diagnosis: as shown in Table 1, HSE with secondary AE and positive CASPR2 and AQP4 antibodies was diagnosed. Taboos were excluded, and the patient’s family agreed to immunoglobulin and methylprednisolone immunomodulatory therapy, acyclovir antivirus, mannitol dehydration, reducing intracranial pressure, improving circulation, nutrition nerve, and other symptomatic support therapy. After 2 weeks of treatment, the patient’s condition improved, and the patient was discharged from the hospital. No discomfort was reported after half a year of follow-up.

**Table 1:** Investigation of clinical features and diagnosis.

Gender	Age	Principal complaint	History	Symptoms	Specialist physical signs	Cerebrospinal fluid	Skull MRI	Detection of macrogenes in cerebrospinal fluid of pathogenic microorganisms	Detection of autoimmune encephalitis-related antibodies and central nervous system demyelinating disease-related antibodies	Detection of tuberculosis, systemic lupus erythematosus, and rheumatoid-related antibodies
Male	14 yr	Headache, dizziness, and fever for 4 days	History of Guillain–Barre syndrome	Fever, headache	Drowsiness, GCS score: 15 points Neck ankylosis left upper limb muscle strength grade 5, left upper limb tendon reflex (+)	Transparent, colorless, positive for Pan test (−), white cell count 332 × 10^6^/L, neutrophil 5%, red blood cell number 1 × 10^6^/L.	Lesions of right hippocampus, amygdala, and insular lobe, local sulcus enhancement of right insular, temporal, and frontal lobes, obvious enhancement of FLAIR, more consideration of meningoencephalitis	Human herpes virus type 1 was detected. Drug-resistant genes of bacteria, fungi, Mycobacterium tuberculosis, parasites, mycoplasma, chlamydia, and bacteria were not detected	AQP4 antibody and CASPR2 antibody IgG were positive	Negative

AQP4 = aquaporin 4, CASPR2 = contactin-associated protein-2, CNS = central nervous system, FLAIR = fluid-attenuated inversion recovery, GCS = Glasgow, HSV = herpes simplex virus, MRI = magnetic resonance imaging.

## 3. Discussion

Fever, headache, and meningeal irritation are the primary clinical signs of viral meningitis, a group of acute meningeal inflammatory diseases caused by various viral infections. The patient in our study had a headache, fever, and neck ankylosis. MRI showed lesions in the right hippocampus, amygdala, and insular lobe, and local sulcus enhancement in the right insular, temporal, and frontal lobes. Cerebrospinal fluid macrogene analysis revealed human herpes virus type I. The patient was finally diagnosed with HSE. HSE is a common fatal sporadic encephalitis in humans that accounts for 20% of all encephalitis cases. The occurrence of HSE is not only associated with the structural characteristics of the virus but also with the tolerance and resistance of the host to the virus. When the function of the host immune system decreases, it can cause the virus to invade the host nerve or activate the virus latent in the nerve.^[4,5]^

Clinical studies have detected other cell surface markers in the cerebrospinal fluid of patients with recurrent or worsening HSE. However, most cases involve n-methyl-d-aspartate encephalitis.^[6,7]^ The anti-CASPR2 antibody was a cell surface protein in the cerebrospinal fluid of this patient. CASPR2 is a cell adhesion molecule and a membrane surface protein expressed in both the central and peripheral nervous systems. Furthermore, it is a voltage-gated potassium channel autoantibody.^[8]^ CASPR2 forms a complex with contact protein-2 to promote the accumulation of potassium channels in the diagonal myelin axon, avoid repeated discharges of neurons, and stabilize the resting potential of the cell membrane. Anti-CASPR2 antibody blocks the formation of the complex by binding to the extracellular segment of CASPR2, depolarizing the cell membrane, and resulting in repeated discharge of the cell membrane. Voltage-gated potassium channels are crucial ion channels that regulate neuronal action potentials and their localization is critical. These disorders can cause seizures, cerebellar ataxia, encephalitis, neuropsychiatric symptoms, and other clinical manifestations. The fact that 84.81% of the patients with anti-CASPR2 antibody-associated encephalitis in the literature were male suggests that it may be caused by the storage and release of the antibody by the male reproductive system. Studies have demonstrated that after viral infection in the CNS, some patients develop parainfectious AE.^[8–11]^ AE occurs in 27% of patients.^[12]^ In this case, the patient first showed signs of herpesvirus encephalitis, was later found to have a positive CASPR2 antibody, and was subsequently considered to have an infection due to AE.

The anti-CASPR2 antibody is not antigen-specific.^[13]^ AE, which is positive for multiple neurons, has gained increasing attention with the expansion of the antibody spectrum. Currently, AE with positive anti-LGI1 antibody and anti-CASPR2 antibody has been found.^[14,15]^ However, the positive anti-CASPR2 and anti-AQP4 antibodies, in this case, have not been reported in the literature to date. Continuous recognition and activation of autoantigens lead to a chronic immune response, accompanied by the production of antibodies targeting different dominant epitopes within the same antigen or between different antigens.^[16]^ It is believed that patients with >1 autoantibody positivity have a more disordered immune system. Studies have shown that, although there are multiple antibodies, the primary symptoms are similar to those of positive antibodies alone, and their clinical characteristics depend on the antibodies responsible.^[11,16]^ Therefore, treatment with the antibody was chosen. The patient was treated with immunoglobulin and methylprednisolone, based on antiviral treatment with acyclovir. Six months after discharge, the patient was followed up in a stable condition.

### 3.1. Strengths and limitations

#### 3.1.1. Strengths.

This is the first reported case of AE with positive CASPR2 and AQP4 antibodies on the basis of HSE. This case will raise awareness of AE with positive CASPR2 and AQP4 antibodies on the basis of HSE, strengthen diagnostic capacities, and provide advice to treat it.

#### 3.1.2. Limation.

Some researchers believe that immunoglobulin is ineffective in treating anti-CASPR2 antibodies (AE).^[17]^ Therefore, individualized analyses should be considered during treatment.

## 4. Conclusion

This study first introduces and analyzes the diagnosis and treatment of AE patients with positive anti-CASPR2 and anti-AQP4 antibodies based on HSE, thereby improving the understanding of the developmental mechanism, treatment, and prognosis of these patients.

## Acknowledgments

This work was supported by the Major Science and Technology Special Project of Yunnan Province [Grant No. 202102AA100061], the Nature Science Foundation of China [Grant No. 81960350], Basic Research of Yunnan Neurological Disease Diagnosis and Treatment Center [Grant No. ZX2019-03-05], Yunnan Health Training Project of High-Level Talents [Grant No. H-2018058], and the Yunnan Applied Basic Research Project-Union Foundation of China [Grant No. 202201AY070001-091].

## Author contributions

**Conceptualization:** Lin-Ming Zhang, Huan-Bo Zhang, Yong-Fang Zou, Ming-Wei Liu.

**Data curation:** Lin-Ming Zhang, Huan-Bo Zhang.

**Formal analysis:** Yong-Fang Zou.

**Investigation:** Huan-Bo Zhang, Yong-Fang Zou, Ming-Wei Liu.**Project administration:** Huan-Bo Zhang.

**Resources:** Yong-Fang Zou, Ming-Wei Liu.

**Software:** Lin-Ming Zhang, Huan-Bo Zhang.

**Supervision:** Yong-Fang Zou, Ming-Wei Liu.

**Writing – original draft:** Lin-Ming Zhang.

Writing – review & editing: Ming-Wei Liu.
